# Mississippi State Dental Association, 1893

**Published:** 1893-06

**Authors:** J. M. Walker


### PROCEEDINGS.

### Mississippi State Dental Association, 1893.

Reported for the DENTAL REGISTER by Mrs. J. M. Walker.

The Mississippi State Dental Association held its nineteenth annual session in the Senate chamber of Jackson, the State capital.

The Association was welcomed by the Mayor of the city, response being made by Dr. Jas. B. Askew, one of the organizers of the Association, its first Secretary and Treasurer and its second President.

Dr. A. A. Dillehay, Meridian, President of the Association, then delivered the customary

### ANNUAL ADDRESS.

Dr. Dillehay expressed his views boldly and fearlessly on some of the important questions now agitating the dental profession. He said that he had been accused of riding some hobby at each meeting of the Association. Admitting the fact, this time his hobby would be surnamed "Dental Ethics." He wished that every dentist would read and re-read the code of ethics until it became so impressed upon him that every violation would gnaw at his conscience until repetition should produce reformation. He hoped that no member of this Association would allow our banner to be trailed in the dust, tattered or torn by casting aside honor and pride for greed of filthy lucre. He called especial attention to that section of the code relating to public advertisements, calling attention to peculiar styles of work, lowness of price, special modes of operating, or claiming superiority over other members of the profession; and to another section, classing as unprofessional and dishonorable, a departure from the rules establishing a standard of fees in any given locality. He spoke of "cutting prices" as an admission of inferiority and inability to cope with one's neighbor. "We are valued at our own valuation." As a rule the advertising dentist is the uneducated dentist, though some who have drunk at the fount of knowledge are so dead to shame, so lost to honorable impulse, so debased in principle as to disgrace their Alma Mater for filthy lucre's sake. He quoted from the annual address of the President of the Louisiana State Society in 1886:

"The advertising dentist is not a modest man, for he does not hide his light under a bushel. He is not a contented man, for he publishes to the world that a generous public has not awarded him his share of employment. He is not an upright man, for he does not obey the code of ethics of his profession. He is an unprincipled man, for he tries to raise his own status by belittling the standard of his fellow practitioners. He is an arrogant man, for he knows it all. He is a conceited man, for he judges others by his own standard. He is a selfish man, for his whole soul is wrapped in self. He is a dishonest man, for he promises results which he knows he cannot attain."

Dr. Dillehay suggested the appointment of a committee on grievances and complaints, whose duty it should be to investigate all cases of violation of the code of ethics on the part of members of the Association, and if not able to bring about a cessation of the same, to report the matter to the Association for action.

He urged the formation of local societies, governed by the code of ethics of the State Association as likely to result in benefit both to the State Association and to the profession at large. He would have it made the duty of such local societies to report to the grand jury, or the district attorney, all known violations of the dental law. To purge the State of empirics and impostors, each member of the profession should constitute himself "a committee of one" to aid the Board of Dental Examiners in enforcing the law. He spoke at some length on the World's Columbian Dental Congress, setting forth the untold benefits to result to all, and expressing the hope that Mississippi would rally to the front.

In the afternoon session the roll of Sections was called, but the chairmen not being ready with their reports, methods for securing papers were discussed. The President stated that he had expected much from the plan decided upon at the last meeting, viz: that the chairman of each committee should consider himself the magnet on that branch, and at an early date furnish the other members of his committee with an outline or abstract of his paper, that they might be prepared to intelligently discuss the paper. He had been promised eight papers for this meeting and felt certain that some of them would be forthcoming.

Dr. J. D. Killian, Greenville, read a paper entitled "A Plea for Physiology and Histology." He said that while his paper was in a measure theoretical, the present age is pre-eminently practical, no quarter being given to theories unless they can be made productive. This is an age of industrialism; in dentistry we have advanced rapidly in manipulative measures, and have brought the mechanical aspect of dentistry to a high state of perfection. But this is not enough. A higher standard requires a thorough and practical knowledge of the functional activities of man and his structural development, through the study of physiology and histology. The average student of a dental college studies these branches merely because the faculty requires it, not that he ever expects to reap any practical benefit from it in actual practice. But these are the windows through which we inspect the functional activities of man. The comparative study of man is now recognized as the only scientific method. Prof. Jos. Le Conte says: "Man is but a part, a very insignificant part of nature, and connected in the closest way with all other parts, especially with the animal kingdom. Man's body is identical with all nature in its chemical constituents; with all vertebrates, especially mammals, in its structure." "There must be breadth of knowledge as well as depth, and to secure breadth we must study collateral branches, as exclusive attention to one thing will make a deep student, but a narrow mind." The scientific study of dentistry should begin at the very foundation of organism, studying the human body in all its different stages and activities. Without this we may be dental laborers, dental workmen, now doctors of dental surgery, without a scientific knowledge of the structure and physical activities of man. Dental college graduates are entitled to administer general anæsthetics, yet how many know the normal rate of heart beats, or pulsations? How many can explain the action of chloroform, ether, nitrous oxide or cocaine, and by what means they cause insensibility? How many can tell the relation the fifth pair of nerves bears to those governing the heart's action, and why the danger incurred in giving an anæsthetic is greater when *they* are involved in the operation to be performed? It is the duty of every dentist to do all that he can to alleviate pain, and he should be competent to employ every means known to medical and dental science. For that reason alone, if for nothing else, we should understand the physiological and structural development of man. A knowledge of histology is of equal importance. We speak glibly of the absorption of the roots of the deciduous teeth, but how many can explain the nature and functions of giant cells, or the different processes by which nature removes from the body "that which cannot be made useful and does not participate in its functions?" We know that we invite structural changes by the premature extraction of deciduous teeth, and thereby promote irregularities, yet how many of us shirk all responsibility and perform the operation that will most readily free us from immediate difficulty regardless of remote consequences. It is cowardly to extract teeth simply because success in other methods is doubtful. We have perhaps arrived at the pinnacle of achievement in mechanical dentistry, but it behooves us to do more to save the natural teeth, and to do this we must familiarize ourselves with their structural development. There is a vast field for improvement before us, especially from a theoretical and scientific standpoint. Anatomy with physiology and histology form the bed-rock on which we should lay the foundation stones of success as scientific practitioners of conservative dentistry.

In the discussion of this paper Prof. Peabody, Louisville, Ky., said that he found physiology, and especially histology, to be the most ungrateful subjects for the teacher. They are difficult to understand and the student is too apt to give his attention in a perfunctory manner, with the idea that the knowledge thus forced upon him will never be of any practical use to him. They say "I don't see what the physiological structure of a tooth has to do with filling a cavity, or making an artificial denture. I have got my sheepskin and I am satisfied."

But while it is true that a skillful operator may by mere mechanical manipulation make a finer gold filling, yet in pathological conditions they are working entirely in the dark.

Dr. W. H. Morgan, Nashville, Tenn., said we practice the healing art very much in the dark unless we know something of pathology and histology. I do not say we must understand them *perfectly*, for I believe that the best of us know only some leading features. But we know something of how tissues are built and consequently something of how they are unbuilt. Before we can learn the nature of disease we must know its pathology, and to know the function of an organ we must know its histology. Dr. Morgan spoke at some length of nature's modes of disposing of foreign bodies. Of the mode of encysting he said that nature builds up around them a shell of connective tissue, of fibrous character and low in organization. This low grade of fibrous encysting tissue is not irritable nor subject to inflammation, and has an important function. In a tooth which has a large abscess at the apex of the root, the bony structure around the tooth is broken down, liquefied, and carried off, making room for the abscess sac, which is sometimes of very considerable size. When we come to the assistance of nature and remove the cause, curing the abscess, nature then forms this abnormal, non-irritable tissue about the root of the tooth, encysting it, so that it may be retained for a long time without giving any further trouble. Dr. Morgan also believes that all the successfully implanted teeth (by the Younger method) are held in place by this tissue forcing its way into all the interstices of the tooth and into the canaliculi, hugging them so closely that they are held firmly in place. This is the only part of the human system where the contact of the soft tissues with devitalized tissue is tolerated. The vitality of this fibrous tissue is so low that it tolerates the presence of the dead end of a tooth when the periosteum has been destroyed.

Papers by Dr. Geo. B. Clement and Dr. W. E. Walker were listed, as they were both detained on the Examining Board.

"Incidents of Office Practice" were next related and discussed.

Dr. K. S. Moffat asked the opinion of the Association in the case of a young girl not yet sixteen years old; the second bicuspid is badly decayed, the cavity running up under the gum; the nerve was exposed and bleeding. This he removed, and filled the root-canal and crown with oxy-phosphate to await his return from this meeting. For permanent work the parents want gold or nothing. He asked if it would be good practice to extract the tooth trusting that the space would close up, or would it be wise to fill such a frail tooth with gold?

Dr. Morgan spoke of the necessity we are sometimes under of deferring to the inclination or requests (not the judgment) of our patients, and of thus working against our own best judgment. In the present case the space would probably close, but at the expense of a swinging around of all the anterior teeth, obliterating the median line thereby giving an unpleasant expression. The choice of alternatives seemed to rest between, 1st and best, an oxy-phosphate filling, carefully watched, and to refill when necessary; 2d, an all-gold crown covering the cavity filled with cement and passing up under the crown. As the patient desires a large gold filling, she would probably not object to this. 3d, A Richmond crown, offering a better appearance, but necessitating cutting off the crown and banding. The last choice would be extracting.

Dr. T. C. West narrated the case of a patient twenty-three years of age, whose teeth up to that time had been in unusually good condition. At that age she noticed a slight overlapping of one of the central incisors. Six months after it was much more decided and one of the centrals devitalized.

On removing the nerve the root was found very much absorbed with a large, open foramen. He filled the root-canal to the best of his ability, but the overlap continues to increase.

Dr. Morgan thought that this was probably a case of incomplete root development, rather than absorption. On one occasion he had removed a devitalized central incisor and found that the root was not a quarter of an inch long, and with a wide, open foramen.

Dr. West had extracted the central incisors for a lady fifty years of age, finding the roots not more than a quarter of an inch long, but well rounded at the apex, and with living pulps.

At the evening session of the first day Prof. Peabody, Louisville, Ky., an honorary member of the Association, read a voluntary essay entitled "The Conservative Treatment of Teeth by the Gold Process."

Prof. Peabody spoke of the supremacy in which gold is held as a filling material, and deprecated the use of gold in teeth of a soft, chalky character, or those attacked by white decay. In those cases he doubts that gold will conserve the tooth or check its disintegration for any length of time. He would not use gold when the adjustment of the rubber dam is very difficult; when the length of time necessary for the insertion of gold would cause such a tax on the patient's strength as to compel us to hurry our work; or in proximal cavities in the posterior teeth where the mouth is so small that it is impossible to gain proper access. In these cases, he asked, is not a perfect filling made from a baser material better than an imperfect filling of the best material? He asked why is it that the use of tin in proximal cavities has been so largely given up? Its conservative character has never been denied, and it must be either because we cannot obtain a sufficiently remunerative fee, or because we fear that other members of the profession, seeing our work, may conclude that we were not equal to the finer metal! When tin does not have to bear the friction of attrition or mastication, it is the equal if not the superior of gold as a conservative filling, particularly in teeth of a soft structure.

Where gold is admittedly the best material for the case in hand, what kind shall we use? Many operators claim that cohesive gold is the only form by which perfect results may be obtained. But may not their failures with soft gold be perhaps due to faulty manipulation? A choice between various materials and various methods may often save from failures and mortification.

Prof. Peabody made the plain statement (though, as he said, expecting antagonism) that soft or non-cohesive foil has more saving properties when introduced into a cavity in a tooth than cohesive gold. While he admitted the value of cohesive foil in its proper place and that in some instances it can accomplish that which cannot be done with any other foil, yet he claimed that it is less kindly received by the enamel and dentine; less in harmony with tooth structure; that it is more liable to be broken down, and that it cannot be brought into as close contact with the walls of a cavity as foils that depend on other principles than cohesion for retention. Non-cohesive foil may be employed by several methods, as on the wedge principle, or on the principle of interlocking or dove-tailing, or by molecular cohesion through a rotary movement of the instrument. Foil that depends on these principles can be forced to the point desired, and pressure on its surface is felt through its whole substance, additional blows only driving it more closely to its place. Cohesive foil is less tractable; it must be placed just where it is wanted, for it cannot be driven; pressure, instead of being felt through its substance, only serves to harden its surface, while if it is not in absolute contact with the walls of the cavity blows on the surface will not make it hug them more closely. In short, soft foil can be made to fit the cavity more closely, to keep out moisture and microbes, and prevent the recurrence of decay at the margins of the cavity. In the approximal surfaces of the posterior teeth, tin or alloy is probably better than gold at the gingival margin. No pits or retaining points are necessary to start the filling, which it is always better to dispense with. It is no lazy man's work to use non-cohesive foil skillfully. It requires strong muscular exertion. A filling of cohesive foil will remain in the cavity with more certainty, but the soft foil filling will be more conservative, and can be introduced in less than half the time. Cohesive foil will undoubtedly save teeth, but it requires a greater degree of patience, a longer expenditure of time and more prolonged physical effort. Fillings of soft gold, the softer the better, made forty or more years ago, are in many cases still standing, preserving the teeth, monuments to the skill of the workers of the material then almost exclusively used.

Why do fillings of gold fail? From lack of judgment as to where gold should be used, and as frequently from lack of skill in using it.

Gold must be so manipulated that it will never, under pressure, draw away from the margins of the cavity. This can be accomplished by always keeping the filling higher in the center than at the edges. In the anterior teeth, in proximal surfaces, the inner portion of the filling should always be in advance of the external, that there may not be a pit at the cutting edge which it is almost impossible to fill. If the cavity cannot be kept perfectly dry, a conservative filling can still be made of non-cohesive foil in the form of cylinders. Passing gold through the flame of a lamp should be avoided, as an excess of heat tends to make it harsh and less yielding. The alloys are unquestionably of value as conservers of dentine if used properly. As to the best way of using them there is undoubtedly much yet to be learned. One says use as dry as possible, another says too little mercury impairs their value. Whatever the manner of mixing or of manipulation, they undoubtedly do prevent decay at cervical walls, and in repairing gold fillings they are better than gold itself. Before the days of alloys or amalgam there was no known way of saving badly broken down teeth. To-day, compar atively useless shells are made serviceable for years, thanks to amalgam. The want of harmony between gold and certain portions of the calcific structure of the dental organs may be due either to the action of the fluids of the oral cavity, or to imperfect adaptation of gold to marginal walls. Want of cleanliness enters largely into these conditions, though if this were the only cause the same results would follow from the introduction of any other metallic filling. An eclectic practice will make us more liberal in our views and tend to give our patients the best services.

Dr. Wm. Crenshaw, Atlanta, Ga., opened the discussion of this paper, which he pronounced conservative, and correct in many of its positions. As a whole the paper is a sort of indirect attack upon the use of cohesive gold. A large proportion of cavities can be perfectly filled only with cohesive gold, and in one-half the time that similar work, if possible at all, could be done with non-cohesive gold. Nothing but cohesive gold can restore incisors that are wasted on the palatine surface and cutting edge, where the gold has to be started on one side and carried over the cutting edge to the other side. Gold will not preserve such teeth if not cohesive. Soft, chalky teeth should not be filled with gold. There is some kind of therapeutic action where tin is put against the walls of such teeth. The amalgam alloys are indispensable. I believe with Dr. Flagg, that in proportion as teeth need to be saved, gold is the poorest material for them. In posterior cavities I would use amalgam properly manipulated. The cements are good only for temporary work.

Dr. W. H. Morgan disagreed with the writer as to the use of gold in soft teeth. He never saw teeth of this character break down under gold that he did not feel satisfied that they would have failed just the same with any other filling material (except the cements which are more closely allied to tooth structure). Dr. Morgan said that he never hesitated to use gold in soft, or so-called "chalky" teeth. Forty years ago he repaired and filled cavities in the proximate surfaces of children not ten years old that are now good teeth. Failure is not the result of want of harmony, or of incompatibility; the fault lies in manipulation. He who uses non-cohesive gold will have a larger proportion of success than he who uses cohesive gold. The only virtue in tin lies in its easy adaptation. It has no therapeutic action. It does not possess antiseptic properties nor does it neutralize acids. Fillings of tin foil do fail at the cervical margins. Cohesive gold has a tendency to draw away from the walls of the cavity; burnishing down at the margins excludes the enemy, moisture, and preserves the tooth, but it does not fill the cavity. Gold should not be annealed in the flame of a spirit lamp. Place it in the oven of a common cooking stove at 212°, or place it in a saucer in front of the fire, and you drive off all surface impurities and get no fumes. It is not necessary to keep your gold or cavity perfectly dry. In a mouth that is moderately healthy there is no disintegrating quality in the oral fluids, and no harm will be done. Neither is it necessary that the filling be hard and dense all through. If the surface is perfectly condensed, and the margins intact, the interior and bottom of the filling may be soft and loose within. If a very large filling is made too dense and hard, the unequal expansion of metal and tooth substance may split the tooth in two.

Dr. Geo. W. Rembert, Natchez, said that he had combated these same points in a similar discussion ten years ago, and had seen no cause to reverse his judgment.

In closing the discussion, Dr. Peabody illustrated many of his positions in regard to shaping cavities and introducing gold, as stated in his paper, by off-hand drawing on the blackboard, without which the points cannot be made clear.

Dr. C. W. Robinson, Magnolia, read a paper on the "Status of Dentistry in the State of Mississippi, under the Present Dental Laws." He asked, is it a profession? or is it a trade? Are we specialists in medicine and surgery? Are we mechanics? or are we nondescripts? In 1878, at a session held in Richmond, Va., the American Medical Association declared by unanimous vote that dentistry was a specialty of medicine and surgery, and that all dentists practicing under diplomas from any reputable college of dentistry were entitled to the rights, benefits and privileges derived from their association with doctors of medicine, thus admitting the importance of this branch of surgery.

Following up this principle, we find at the present day that many of the medical colleges have adopted their dental departments with a full corps of teachers, where the student of dentistry shares alike with the student of medicine in all that pertains to either, without distinction or discrimination, the civil law also making no distinction. In the State of Mississippi, however, Dr. Robinson pointed out that there is a marked discrimination in favor of the medical practitioner, which he considered unjust, humiliating and inconsistent. As an instance of this he cited the fact that while both medical and dental practitioners, even though holding well-earned diplomas, are required to present themselves before a Board created by the law-making power, and undergo a rigid examination, the law thus far very properly regarding the dentist in the same light as the physician—proficient in those studies which make them both competent to practice medicine, perform surgical operations, or give advice by which a diseased part of the human body may be restored to health, and the people protected against charlatanism. But, with strange inconsistency, while the physician and dentist are alike admitted to practice only under certificates granted by their respective State Boards, paying the same fee, yet the physician, by virtue of his license, is exempt from the payment of privilege tax, while the dentist though armed with a similar license is required to walk up and settle his privilege tax like all other *tradesmen* and *mechanics*. The law placing the dentist in that category, while the physician is omitted. Again, all *physicians* in active practice are exempt from serving as jurors in courts of law, but the *dentist* is only exempt when through the courtesy of the judge he is excused. While the M.D. sits quietly in his *sanctum sanctorum*, or rides over the country visiting his patients, the D.D.S. must close his office or turn it over to an assistant, his patients left to endure as best they can the pangs of aching teeth while the poor D.D.S. in charge of a bailiff is locked up in a dirty, ill-ventilated jury-room of some county court, wrestling manfully with the momentous question as to whether "Sambo killed the hog, or did it die a natural death?" Is this right? Is it just? We are either specialists of surgery and medicine, or we are mechanics. If the former, by virtue of the diploma which we hold as Doctors of Dental Surgery, we are justly entitled to all the rights and privileges enjoyed under the law by the general surgeon and physician. If the latter, then the D.D.S. has no meaning and should be discarded—the State Board of Examiners discarded as superfluous, allowing every individual to seek such distinction as his skill and talent may allow. But I believe it is a duty which we, as an Association, owe to the profession, that we should earnestly insist that the Attorney General of the State construe the law and define our status under that law. Should that learned gentleman decide that in his opinion we, under the present law, are to be classed with mechanics and tradesmen, then we should memorialize our legislature to enact such laws as will place the doctor of dental surgery on the same plane as the doctor of medicine and general surgery. Not until that is done can the D.D.S. command the same consideration and respect as the M.D.

The suggestions offered in this paper were discussed with great animation by Drs. J. B. Askew, T. O. Payne, W. E. Walker, J. O. Frilick, W. H. Morgan, Geo. W. Rembert, F. Peabody, W. T. Martin, I. B. Rembert and others, as the outcome of which, a standing committee on dental legislation and charter was formed, whose duty it was made to obtain the construction of the present law from the Attorney General; to frame a bill eliminating the objectionable features of the existing law, to be presented to the Association at its next meeting, and also to obtain a charter for the Association as promptly as can be done in conformity with the requirements of the law.

Dr. I. B. Rembert, Jackson, chairman, with Drs. Askew, Luckie, Robinson, Marshall and Clement were appointed on this important committee.

The second day of the meeting was devoted entirely to clinics.

The morning session of the third day the troublesome question of *arrears of dues* was discussed. All members in arrears for three years having been notified by the Secretary, after the last meeting, that, in accordance with a resolution previously adopted, their names would be dropped if remittance was not made, those who had made no response were ordered dropped from the roll, with the privilege of coming in again as new members if satisfactory explanation was furnished the committee on membership.

The Board of Dental Examiners being still in session, a paper by Dr. W. E. Walker, entitled "Three Remedial Preparations", was read and discussed in his absence. In this paper the claims made by the manufacturers of "Pyrozone," "Weld's Syrup of Iron Chloride," and the "Iron Bi-palatinoids," were set forth, with a brief abstract of the published observations of Drs. Ottolengui, Boedecker, Chas. B. Atkinson, M. L. Rhein and others with confirmation as far as gained in his own experience. Dr. Walker spoke of the unreliability of Peroxide of hydrogen, from its lack of stability. In the three grades of Pyrozone (1) medicinal, (2) antiseptic, (3) caustic, we seem to have reliable, stable preparations of oxygen in solution of exact strength, which, in the grade indicated by conditions, will be found very valuable: (1) in the diagnosis of pus, (2) in the treatment of ulcerations, dental abscesses, pyorrhea alveolaris, necrosis, (3) in bleaching teeth, (4) as a cleanser, (5) as a disinfectant, (6) as a hæmostatic. Medicinal Pyrozone is a 3 per cent. or fifteen volume aquæous solution $H_2O_2$, Antiseptic Pyrozone a 5 per cent. solution $H_2O_2$ in ether, Caustic Pyrozone a 25 per cent. solution $H_2O_2$ in ether. Medicinal Pyrozone has about the same range of application as Peroxide of Hydrogen, but with the advantages of absolute stability and exact strength. Its claims are that of being a rapidly-acting, non-toxic, non-acid, odorless antiseptic and pus destroyer. It may be freely and beneficially used as a mouth wash.

Dr. M. L. Rhein says: "It does not seem to lose its stability after any length of time."

Antiseptic Pyrozone acts on pus with great energy, the rapid evaporation of ether leaving the concentrated $H_2O_2$ behind. An apparent coagulation follows contact with soft tissues, but there is no permanent change of tissue; the natural color soon returns, with no eschar or slough. With care in avoiding exposed pulps it is very valuable in spraying cavities when ready for filling, sterilizing and drying the cavity at the same time. The Caustic Pyrozone is pronounced by Dr. Chas. B. Atkinson "probably the best bleacher of teeth that has ever been offered; * * * its effect is exceedingly prompt, and the results permanent." In deep pockets of pyorrhea alveolaris a small tent with the 25 per cent. will in most cases terminate the suppuration.

Tannin and glycerine promptly relieve the pain and burning consequent upon accidental contact with healthy soft tissues, which is more severe than upon diseased tissue. As a *pus indicator*, it must be borne in mind that effervescence and froth follow its contact with all extraneous organic matter, whether pus or a flow of venous blood. Pyrozone is an absolute indicator of pus, then, *only* after the territory has first been bathed and rinsed and made clean.

In "Weld's Syrup of Iron Chloride" it is claimed that the disastrous effects of the tincture upon the teeth have been entirely overcome, the free acid being neutralized while the therapeutic value remains unimpaired.

In the bi-palatinoids the agents to be combined are contained in two halves of a double-convex capsule, but kept apart by a soluble air-tight partition until the capsule is dissolved in the stomach when chemical or molecular reaction takes place. In the *Ferrous Carbonate* bi-palatinoids, for instance, 1 gr. pure desiccated powdered sulphate of iron, and $\frac{2}{3}$ gr. carbonate of soda are enclosed, each in an air-tight compartment of the double or twin capsule. When this is dissolved in the stomach molecular reaction takes place and the green ferrous carbonate results, the chemical form of iron most easily absorbed by the system, no inert substance being taken into the stomach.

Dr. C. B. Atkinson considers this method of administering iron tonics far superior to any liquid preparation.

In the discussion of this paper, Dr. Morgan said that he had not been carried away by the peroxide of hydrogen craze, as he had not been able to obtain the results claimed for it by others. He had not used Pyrozone, but thought that the effervesence resulting in the use of these agents was due solely to sulphuric acid in the preparations. If Pyrozone is a stable preparation it will be preferable to peroxide of hydrogen.

A strong spray of clear water will cleanse an ulcerated surface better than can be done by any other means.

If it removes the discoloration of dentine it will be a valuable agent, and by its power of penetration it will reach the debris in the tubuli.

It is a mistake to claim that it is an absolute destroyer of bacterial life. Prof. Miller, of Berlin, has proven that nothing as yet known has that power. Carbolic acid is the most effective agent in that direction, as it is an active coagulant, preventing decomposition through its power of coagulation.

Dr. Morgan said the paper is worthy of attention and deserves a place in our literature. "Some day I hope to see it in print, as it requires investigation and study. Then I may have more to say about it."

Dr. W. T. Martin spoke of the properties of sodium peroxide, as recently set forth by Dr. E. C. Kirk.

Dr. Peabody said that he was not a chemist, but had been a careful observer of the effects of the different remedial medicinal agents, and had noted the difference between theories and the facts observed in practice. He had not as yet used Pyrozone. He said, "I do not believe that Peroxide of hydrogen ever destroys pus. It is neither a disinfectant nor a germicide. Effervesence is no proof of pus, as we get the same froth from mucus or from venous blood."

Dr. Peabody said that last year at different meetings he had taken some pains to set forth the merits of iodoform vapor, injected into root-canals, having investigated it with great care. It penetrates the dentine, and the precipitate from the vapor makes an invaluable filling at the apex of the root. It may not destroy microbes, but if you put an antiseptic in bacteria will keep away. It is true there is no medicine that will kill the spores—the ova—they must be hatched before you can kill them. There is nothing that can be applied in the human system that will destroy them: boiling water cooks the germ and effectually disposes of it. He had received fromParke, Davis & Co. a sample of "Weld's Syrup of Iron Chloride," and had read the literature accompanying it with care and interest. He filled a small bottle with half-and-half chloride of iron (Weld's syrup?) and water, and placed a perfect bicuspid tooth in it. A cloudy precipitate formed, but there was no decalcification of the enamel. He then placed another tooth in a bottle of full strength. At the end of twelve hours there was such a heavy cloudiness that the tooth could not be seen in the fluid, and the enamel was much roughened At the end of twenty-four hours more the enamel at the gingival margin had disappeared. At the end of another twenty-four hours the enamel was off in spots on the buccal and lingual surfaces.

Physicians advise their patients to take chloride of iron through a glass tube, but that will not keep it off. They should instruct them to wash the mouth and teeth with an alkaline wash, to neutralize the hydrochloric acid and prevent its injurious effects upon the teeth.

Dr. Morgan thought the ordinary tincture of iodine would have the same beneficial effect as the iodoform vapor, penetrating the dentine quite as effectually without the very offensive odor of iodoform.

Dr. J. B. Askew said that he had not had the opportunities for experiments enjoyed by the professors in dental colleges who had spoken on this subject, but that he had never deserted his first love, carbolic acid.

Subject passed.

The clinics of Thursday were placed under discussion.

Dr. Geo. B. Clement illustrated his simplified method of making a metal crown using a platinum band with gold masticating surface. He claims for this method that the work of soldering is much simplified and facilitated while the crown is less conspicuous than all gold.

Dr. Geo. W. Rembert thought that though crown work was not usually attempted by beginners, yet this would be found one of the easiest methods.

Dr. W. H. Marshall having made a large amalgam filling using his matrix, this gave rise to a lengthy discussion on the methods of manipulating the alloys, the value of amalgam as a filling material, and the cause of failures. Nothing new was elicited but the records of experience and observation in the use of this material were interesting and valuable.

The other clinics were not discussed.

Dr. Geo. B. Clement read a paper on "Chemistry." He said that having been placed in the Section embracing chemistry, he had endeavored to do his duty to the best of his ability. As the other members of the Association were probably quite as familiar with the subject as himself, he would endeavor only to place some of the elementary principles of chemistry within the comprehension of the younger members, by which he might possibly accomplish some little good. He said that the absolute certainty of results under given conditions made chemistry one of the positive sciences—whenever the two gases, hydrogen and oxygen are brought together in certain known proportions, we know to a certainty that the liquid water will be the product. While there are pet theories in chemistry as in all other sciences, there is a sufficiency of demonstrated facts to constitute it one of the most perfect and most exact of sciences. To be a thorough chemist demands all of a man's time and all of his labor. We, then, cannot expect to be practical chemists, but one should know something of its teachings, and especially in dental chemistry. Dentistry would be more perfect as a scientific profession if we were better chemists.

Dr. Clement then outlined simply and briefly the fundamental principles of chemistry—its two great subdivisions, organic and inorganic; defined the theory of atomic weights and molecular weight, the derivation of the modern nomenclature with its symbols, prefixes and terminations, so simple and yet so complete, relieving the memory of a herculean task. The few letters and figures combined in the symbol tell the whole story, its origin, nature, composition and proportions. The results of the combination of the various elements are often seemingly most contradictory; thus yellow sulphur and white quicksilver when combined form red vermilion, absolutely unlike either of its two component parts in color, consistency, weight or properties. Solid black charcoal and yellow sulphur unite to make a colorless fluid.

Poisonous and offensive chlorine combined with the brilliant metal sodium forms that common culinary necessity—salt. The more dissimilar the substances the stronger the union. Though such an exact science, chemistry is also an endless experimental science through the peculiar range of chemical affinity. It is only through chemical affinity that we are able to utilize for our sustenance and aid those natural elements most essential to our very existence. Phosphorus for the brain, iron for the blood, calcium for the bones, carbon, oxygen, hydrogen would be locked up unavailable for our use except through the action of chemical affinity, through the agencies of the animal and vegetable kingdoms, those great laboratories of nature. Carbon, hydrogen and oxygen are transformed into starch, sugar, vinegar and alcohol. All that we eat, all that we drink, all that we wear, we owe to the transforming agencies of chemical affinity.

The leaf, the flower, the vine, the tree, are all unceasingly at work for man. God has given to man the materials and the possibilities, but he must work out for himself the usage of everything for his own good. It is this wise provision, this gap in actual knowledge, which has made man the king of creation, in intellect. A benificent Providence gave the elements and fashioned a mind for man with a peculiar instinct of development. So, to-day, through the science of which we treat, as a reward for his labor man has found a remedy for almost every ill, an abundance of food, drink and raiment.

Dr. Dillehay. Although I am not a chemist, yet I consider that the consideration of this subject elevates man in his attitude towards his Creator, the giver of all good.

### AN INTER-STATE DENTAL EXAMINING BOARD.

Dr. W. E. Walker, Bay St. Louis, President of the Board of Dental Examiners, said that during the present meeting of the Board many things had forced the conclusion upon his mind that something is imperatively needed to bring about a greater degree of harmony between the different State Boards, and between the Boards and Colleges. What is needed would seem to be an Inter-State Dental Examining Board supplemental and different in function from the National Board of Dental Examiners. He said that he had not had time or opportunity to mature a plan; the scope of such a Board should be broad and liberal and he would offer as suggestions only, that this Board should properly be composed of one delegate from each State Examining Board that might endorse this plan. A license granted by the Inter-state Board should qualify a man to practice in any State of the Union represented on this Board without undergoing another examination.

The function of this Board would be to examine all applicants for an inter-state license, both in "learning" and "skill" the standard not to fall below that required for graduation by the colleges having the severest requirements, and with the exception of holders of State licenses, none but graduates should be allowed to appear before the Inter-state Board.

A provision might be included which would entitle our best "old men" to an inter-state license without examination, provided they have held a State license for a period of at least five years and properly endorsed recommendations from their State Society and from the existing State Board in lieu of five years' State license, in case such State has not had a State law or State Board for five years at the time of their application. If such a plan could be perfected and put into execution a man could go before such a Board while in his prime, and if he passes the examination, which should always be fully up to the then implicit standard of professional attainment, he would receive a license which would entitle him to a State license in any of the States represented on the Inter-state Board on satisfying the demands of the State law by a formal examination and payment of the legal fee. Dr. Walker illustrated various cases in which ample justice would thus be rendered on all sides and existing discords harmonized.

In the discussion of the plan suggested by Dr. Walker, Dr. Geo. B. Clement, Secretary of the Board, said, I have had some conversation with Dr. Walker about this idea, and I think it is exceedingly suggestive. It aims at the correction of numerous evils arising from our conflicting State laws.

Dr. Dillehay thought that if properly worked out it would correct the injustice of compelling an old practitioner, who has grown gray in the practice of his profession, but perhaps gotten rusty on theory, of having to undergo an examination like a school boy, compelled perhaps by failing health—he can locate in some more congenial climate. He might desire to come among us, but falling below the average required, we lose a valuable accession to our working members. If such men as even Prof. Morgan, or Prof. Peabody, desired to locate in the State of Mississippi, they would have to undergo a Board examination like a lot of raw college boys. This plan of Dr. Walker's looks to the correction of such incongruities.

Dr. Marshall: It would perhaps require some change in the existing State laws, but some plan ought to be perfected by which a man in good standing in one State can be admitted to practice in another State.

Dr. Dillehay: If men of fame and reputation desire to come among us we should feel honored by their coming, and the Board should be competent to license them. I think the Association might instruct the Board to use discretion in such cases.

Dr. Nisbet: An Inter-state license as proposed would be a passport in obtaining a State license. The law requires an examination to prove the applicant qualified to practice dentistry, and to be of good moral character. This Inter-state license would be a guarantee of that. Mississippi, for instance, would have had a representative from her State Board, on the Interstate Board, who would know how he had passed his examination, and what his credentials were.

Prof. Peabody: Dr. Walker has presented a most admirable nucleus on which to build. There is something wrong in compelling a man to undergo a worse than college examination in every State where he may wish to go. He may have devoted a lifetime to the service of his profession, but may not have looked into a text-book for years. We all forget those things of which we never make any practical use.

It is to be regretted that time did not permit of this matter being presented in more perfect shape. The endorsement of such an Inter-state Board would be recognized as evidence of capacity and character. Our profession is a liberal one, and we should eliminate everything that looks only to the benefit of a chosen few.

Dr. W. H. Morgan: I like the spirit of this movement very much. The relations between the Colleges and Boards are not what they should be. They are not yet in harmony. The spirit of this movement is in the right direction.

He said the colleges try to meet the demands of the profession. We say to the Examining Boards, go ahead; put up your standard and we will try and bring our students up to it. We intend to bring our young men up if you don't go too fast. We intend to have them pass right through.

Dr. W. E. Walker: The plan is not yet fully matured, but I think it can be so adjusted as to avoid any necessity for changing State laws. It is sometimes a troublesome thing to get laws changed. In the proposed Inter-state Board, all the States represented would be admitted on an equal standing. In some of the States the law is inadequate and the Board too lenient. Alabama, I understand, has a very strict Board, and would not be willing to have a man holding a license from a Board whose requirements were almost *nil* declared competent to come into Alabama and practice without any further preliminary. But if a man has such an Inter-state license as I propose—the details of which have yet to be perfected, for, so far, this is only suggestion—it would be a guarantee that he is a good and competent man, and a mere formal examination, sufficient to comply with the requirements of the State law, would be all that would be necessary to admit him to registry in the State.

Dr. Geo. B. Clement: I now move that a committee be appointed to investigate this matter, and report before the close of this meeting. We must have something to start on, and we would then be prepared to report at the meeting of the National Board of Examiners at Chicago this summer. I would like to have the representatives of our State Board go prepared to present the views of this Association before the National Board.

The Chair: This matter is rather deep to be brought up on such short notice.

Dr. Walker: It is a matter that has been forced on my mind by what I have seen and learned at the present meeting of the State Board, but I have not had time or opportunity to thoroughly mature any definite plan in detail.

No action followed this discussion.

The election of officers resulted as follows:

Dr. Jas. B. Askew, Vicksburg, President; Dr. Geo. W. Rembert, Natchez, 1st Vice-Pres.; Dr. J. O. Frilick, Meridian, 2nd Vice-Pres.; Dr. W. C. Stewart, Fayette, 3d Vice-Pres.; Dr. J. D. Killian, Greenville, Cor. Sec.; Dr. T. C. West, Natchez, Rec. Sec.; Dr. C. C. Crowder, Kosciusko, Treasurer.

Natchez was selected as the next place of meeting, and the first Tuesday in May the time.

The Board meets, by law, in Jackson on the first Tuesday in April.

On motion, adjourned to meet in Natchez on the first Tuesday in May, 1894.

